# Supplementary material for: Artificial intelligence in human resource development: An umbrella review protocol
Source: PLoS One. 2024 Sep 9;19(9):e0310125. doi: 10.1371/journal.pone.0310125 (PMC11383213; doi:10.1371/journal.pone.0310125)
Supplement: S2 File — (DOCX) [file pone.0310125.s002.docx]

**S2. Search term identification strategy**

Given the exploratory nature of this project, we aimed to compile a list of topic-related terms to create a comprehensive search string (see Table 1 in the main protocol). Given the exploratory nature of this project, we aimed to compile a list of topic-related terms to create a comprehensive search string (see Table 1 in the main manuscript of the protocol). To accomplish the goal systematically, we took the following procedure to collect AI-related and HRD-related terms:

First, three authors independently conducted preliminary searches for literature review articles on AI-related technologies in HRD and related areas in Scopus and Google Scholar. The preliminary search aimed to understand and collect basic search terms used in the existing literature reviews on similar topics to our project. Thus, three authors conducted this search to find the review articles published within the recent three years (2021-2023) using a search string with Boolean operators and the common search terms in AI and HRD: (“artificial intelligen*” OR “AI” OR “large language model*” OR “LLM” OR “machine learning” OR "ML") AND (“human resource*” OR “HRD” OR “HRM”). This stage was coordinated using Covidence, and we completed the preliminary search in September 2023.

A total of 27 studies were identified, with 17 duplicates removed from the initial pool of 44 imported studies. Among the 27 studies, eight were excluded based on titles and abstracts. We then assessed the full texts of the remaining 19 studies, leading to the exclusion of three more. The final 16 studies were used to extract AI-related and HRD-related search terms. Table S2 provides an overview of the 16 studies from which AI-related and HRD-related search terms were extracted. This preliminary stage allowed us to build a basic list of search terms for our umbrella review.

Second, as a boundary condition in which AI will be used, we further collected HRD-related search terms from existing articles exploring a knowledge map and research trends in the field of HRD (Han et al., 2017; Shirmohammadi et al., 2021; Yoo et al., 2019; Yoon & Chae, 2022). The trends and/or themes in HRD were scraped as HRD-related search terms for our umbrella review. Additionally, given the recent interest in people analytics in HRD, we collected people analytics and similar terms, such as HR analytics, based on a review article on people analytics (Tursunbayevaa et al., 2018).

As a final step, the search terms identified through the above stages were validated by using VOSviewer (cf. Tuffaha & Perello-Marin, 2021). Using the broad terms “artificial intelligence” and “human resource”, we collected keywords from peer-reviewed journal articles and conference proceedings indexed in WoS Core Collection and Scopus. We then used VOSViewer to identify the most relevant keywords. Specifically, a keyword had to co-occur at least five times to be considered relevant (cf. Kaushal et al, 2023). We compared the 995 unique keywords across the two databases to the search terms identified through the manual process to determine if any keywords should be added. As a result, we have newly added 35 new AI-related search terms and six HRD-related terms to our search strings. Additionally, we have edited 12 existing search terms to ensure comprehensive coverage of keywords. The comparison process and results, detailing keywords from VosViewer and the finalized search terms, are summarized in the supplementary file (S3 file). The S3 file also includes the tab that summarizes the search term history showing which keywords were newly added or edited. The comparison process and results, which outline keywords from VosViewer and the finalized search terms, are summarized in the supplementary file (S3 file). Additionally, the S3 file includes a separate tab summarizing the search term editing history, indicating newly added search terms and edited terms during the VosViewer process.

**References**

Han, S. H., Chae, C., Han, S. J., & Yoon, S. W. (2017). Conceptual organization and identity of HRD: Analyses of evolving definitions, influence, and connections. *Human Resource Development Review, 16*(3), 294-319. https://doi.org/10.1177/1534484317719822

Kaushal, N., Kaurav, R. P. S., Sivathanu, B., & Kaushik, N. (2023). Artificial intelligence and HRM: Identifying future research agenda using systematic literature review and bibliometric analysis. *Management Review Quarterly*, *73*(2), 455-493. https://doi.org/10.1007/s11301-021-00249-2

Shirmohammadi, M., Hedayati Mehdiabadi, A., Beigi, M., & McLean, G. N. (2021). Mapping human resource development: Visualizing the past, bridging the gaps, and moving toward the future. *Human Resource Development Quarterly, 32*(2), 197-224. https://doi.org/10.1002/hrdq.21415

Tuffaha, M., & Perello-Marin, M. R. (2023). Artificial Intelligence definition, applications and adoption in Human Resource Management: A systematic literature review. *International Journal of Business Innovation and Research, 32*(3), 293-322. https://doi.org/10.1504/IJBIR.2023.134887

Tursunbayeva, A., Di Lauro, S., & Pagliari, C. (2018). People analytics—A scoping review of conceptual boundaries and value propositions. *International Journal of Information Management, 43,* 224-247. https://doi.org/10.1016/j.ijinfomgt.2018.08.002

Yoo, S., Jang, S., Byun, S. W., & Park, S. (2019). Exploring human resource development research themes: A keyword network analysis. *Human Resource Development Quarterly, 30*(2), 155-174. https://doi.org/10.1002/hrdq.21336

Yoon, S. W., & Chae, C. (2022). Research topics and collaboration in human resource development review 2012–2021: A bibliometrics approach. *Human Resource Development Review, 21*(1), 24-47. https://doi.org/10.1177/15344843211068807

**Table S2. Overview of the 16 Studies Used for Search Term Extraction**

| **No** | **Title** | **Authors (up to 3 authors)** | **Year** | **Journal** | **DOI** |
| --- | --- | --- | --- | --- | --- |
| 1 | From traditional to smart human resources management | Kambur, E.; Yildirim, T. | 2023 | International Journal of Manpower | 10.1108/IJM-10-2021-0622 |
| 2 | An interdisciplinary review of AI and HRM: Challenges and future directions | Pan, Y.; Froese, F.J. | 2023 | Human Resource Management Review | 10.1016/j.hrmr.2022.100924 |
| 3 | Unlocking the value of artificial intelligence in human resource management through ai capability framework | Chowdhury, S.; Dey, P.; Joel-Edgar, S.; et al. | 2023 | Human Resource Management Review | 10.1016/j.hrmr.2022.100899 |
| 4 | A systematic literature review on the impact of artificial intelligence on workplace outcomes: A multi-process perspective | Pereira, V.; Hadjielias, E.; Christofi, M.; et al. | 2023 | Human Resource Management Review | 10.1016/j.hrmr.2021.100857 |
| 5 | Critical exploration of AI-driven HRM to build up organizational capabilities | Böhmer, N.; Schinnenburg, H. | 2023 | Employee Relations: The International Journal | 10.1108/ER-04-2022-0202 |
| 6 | Intelligent human resources for the adoption of artificial intelligence: A systematic literature review | Jatobá, M. N.; Ferreira, J. J.; Fernandes, P. O.; et al. | 2023 | Journal of Organizational Change Management | 10.1108/JOCM-03-2022-0075 |
| 7 | Artificial intelligence and HRM: Identifying future research agenda using systematic literature review and bibliometric analysis | Kaushal, N.; Kaurav, R. P. S.; Sivathanu, B.; et al. | 2023 | Management Review Quarterly | 10.1007/s11301-021-00249-2 |
| 8 | Decoding ChatGPT: A taxonomy of existing research, current challenges, and possible future directions | Sohail, S. S.; Farhat, F.; Himeur, Y.; et al. | 2023 | Journal of King Saud University-Computer and Information Sciences | 10.1108/CEMJ-02-2023-0091 |
| 9 | Artificial intelligence (AI)-assisted HRM: towards an extended strategic framework | Malik, A.; Budhwar, P.; Kazmi, B. A. | 2022 | Human Resource Management Review | 10.1016/j.hrmr.2022.100940 |
| 10 | Artificial intelligence, robotics, advanced technologies and human resource management: A systematic review | Vrontis, D.; Christofi, M.; Pereira, V.; et al. | 2022 | The International Journal of Human Resource Management | 10.1080/09585192.2020.1871398 |
| 11 | A review of machine learning applications in human resource management | Garg, S.; Sinha, S.; Kar, A.K.; et al. | 2022 | International Journal of Productivity and Performance Management | 10.1108/IJPPM-08-2020-0427 |
| 12 | Artificial intelligence in learning and development: A systematic literature review | Bhatt, P.; Muduli, A | 2022 | European Journal of Training and Development | 10.1108/EJTD-09-2021-0143 |
| 13 | Artificial intelligence in human resources management: A review and research agenda | Gélinas, D.; Sadreddin, A.; Vahidov, R. | 2022 | Pacific Asia Journal of the Association for Information Systems | 10.17705/1pais.14601 |
| 14 | When technology meets people: The interplay of artificial intelligence and human resource management | Qamar, Y.; Agrawal, R.K.; Samad, T.A.; et al. | 2021 | Journal of Enterprise Information Management | 10.1108/JEIM-11-2020-0436 |
| 15 | Artificial intelligence in tactical human resource management: A systematic literature review | Votto, A.M.; Valecha, R.; Najafirad, P.; et al | 2021 | International Journal of Information Management Data Insights | 10.1016/j.jjimei.2021.100047 |
| 16 | Artificial intelligence definition, applications and adoption in human resource management: A systematic literature review | Tuffaha, M.; Perello-Marin, M. R. | 2021 | International Journal of Business Innovation and Research | 10.1504/IJBIR.2021.10040005 |
