# Supplementary material for: Artificial intelligence in human resource development: An umbrella review protocol
Source: PLoS One. 2024 Sep 9;19(9):e0310125. doi: 10.1371/journal.pone.0310125 (PMC11383213; doi:10.1371/journal.pone.0310125)
Supplement: Attachment — Submitted filename: AI_SLR_Protocol_Response Table_r1_final.docx [file pone.0310125.s004.docx]

**Artificial intelligence in Human Resource Development: An umbrella review protocol**

We greatly appreciate the valuable comments. They prompted us to revise our manuscript thoroughly, with the hope of enhancing its overall quality. In this response letter, we have provided our point-by-point explanations addressing each comment by specific areas of focus. In particular, we’ve preregistered this protocol to OSF (<https://doi.org/10.17605/OSF.IO/Z8NM6>).

| **Reviewer#1 Comments** | **Actions/Responses** |
| --- | --- |
| I recommend citing and reviewing the updated PRISMA document, "The PRISMA 2020 statement: an updated guideline for reporting systematic reviews" published in BMJ 2021; 372 doi: . | We appreciate the recommendation. We have updated our reference to include the PRISMA 2020 statement with a clarification of using the updated PRISMA 2020 in the main research using this protocol (p.5 lines:88-91).  “In the main research using this protocol, we plan to incorporate guidelines from the updated PRISMA 2020 statement to ensure comprehensive reporting of our umbrella review (Page et al., 2021).”  Additionally, it should be noted that our first supplement file (S1) is based on PRISMA-P 2015 checklist, because this is for the protocol. |
| Consider supplementing the database search with AI tools such as Perplexity and Consensus. | Thank you for your suggestions. We explored the potential of the integration of AI tools to enhance our database search. However, we have decided not to utilize AI tools such as Perplexity and Consensus for this review. Our focus will remain on traditional database searches. As outlined in our protocol, our study is an umbrella review aiming to offer a comprehensive overview of existing systematic reviews relevant to our research questions. Given our research purpose, this decision ensures that our methodology remains consistent with established practices in systematic reviews and maintains the comparability of our results with other studies in HRD and related areas.  Additionally, while the AI-based literature search tools provide a great alternative to streamline the research process, the technology employed by Consensus, Perplexity and similar existing platforms, although advanced, is still in its early stages. These tools are based on LLMs and the search strategy evolves with time with a high possibility of varying results with time which can cause an adverse effect on replicability.  Reflecting on this valuable comment, we will consider discussing this matter in the future research directions in our main manuscript, stating the potential of AI tools to streamline literature reviews and unlock new levels of efficiency and productivity. |
| I was unable to access the supplementary files, so I cannot provide comments on them. | We were sorry for the inconvenience. To make sure that the supplementary files are accessible in the revised submission, all supplementary files have been uploaded in an OSF repository (<https://osf.io/af6d7/>). In addition, we’ve added the separate links to the individual supplementary files for your reference.  In the main protocol, we have stated it (p.13 line:244).  “**Note**: All supplementary files are available in an open-access repository: https://osf.io/af6d7/” |
| The authors mention that they will use a different program than Covidence for information extraction since it only allows two coders. Please specify which program will be used. | Thank you for noticing it. We have determined to use Rayyan throughout this umbrella review project to ensure replicability of our protocol. We have clarified it in our revision (p.8 lines:133-137)  “We will use Rayyan to extract data. Extraction fields will be set up with the relevant information from the studies, and Rayyan’s tagging and coding features will be used to categorize and organize the extracted data. Disagreements will be discussed and resolved using Rayyan’s conflict resolution feature. The extraction fields for recording the finally selected systematic review studies will include”. |
| It is unclear which descriptive statistics will be used and which programming language will be utilized for this purpose. Is R being used? | We have clarified the use of descriptive statistics and specified the program (i.e., R) (p.10 lines:172-176).  “Descriptive statistics involves quantifying the occurrence of specific themes or concepts related to AI implementation in HRD across the selected systematic literature reviews. (…) R will be utilized for statistical analysis and visualization”. |
| Additionally, will inferential statistics be employed in the study? | Thank you for the question. Inferential statistics are not planned for this umbrella review as the primary focus is on thematic synthesis and descriptive analysis. If necessary, we will clarify this (not employing influential statistics) in the main protocol. |
| Data and all analyses should be placed in a repository and the URL provided in the manuscript. | Thank you for the comment. We will ensure that all data and analyses are placed in a repository. We have clarified this in the revised protocol (p.10 lines:183-184).  “All data and analyses will be placed in an open-access repository, and the URL will be provided in the final manuscript”. |

| **Reviewer#2 Comments** | **Actions/Responses** |
| --- | --- |
| In their work, the authors explored different ways to define research terms based on a initial search with key terms such as artificial intelligence, large language model, machine learning and human resource, from which they extracted more specific terms and also included some trendy terms related with analytics, and after that, the relevance of the resulting terms were validated using the VOSViewer software, to finally propose a search string to developing the review in databases such as Scopus or WoS.  Unfortunately, the proposed protocol describes a search process in which the authors made some decisions that made the protocol very specific, when they included, for instance, terms related with analytics, only because they are trendy. | Thank you for sharing the concerns on our search terms.  However, the keywords, terms related to analytics, were included to ensure comprehensive coverage in our search. Because we treat search terms in the same bucket with the “OR” function, including these keywords (e.g., HR analytics) will not make our search limited.  Also, as we have specified all the process of our systematic review in a transparent way, having these terms do not impact the replicability of our protocol. |
| Another aspect affecting the replicability of the protocol is that the authors do not list, in the references, the 16 studies from which extract AI-related and HDR search terms.  For these reasons, from my point of view, the protocol presented here is not replicable. | Thank you for suggestion.  We have included a list of the 16 studies used to extract search terms in our supplementary file (see Table S2 in the S2 supplementary file). To clearly deliver our protocol, we have separately created the supplementary document (S2) to detail the search term identification strategy for compiling the lists in Table 1 in the protocol.  We have also clarified this in the main protocol (pp.5-6 lines:100-105).  “AI-, HRD-, and SLR-related strings include search terms combined using the Boolean operator OR. In the final search string, the Boolean operator AND will be used to combine the three sub-strings. (…) The supplementary files (S2 & S3) detail the procedure for determining AI- and HRD-related terms in Table 1”. |
