# Supplementary material for: Artificial intelligence in human resource development: An umbrella review protocol
Source: PLoS One. 2024 Sep 9;19(9):e0310125. doi: 10.1371/journal.pone.0310125 (PMC11383213; doi:10.1371/journal.pone.0310125)
Supplement: Attachment — Submitted filename: AI_SLR_Protocol_response_r2.docx [file pone.0310125.s005.docx]

**Artificial intelligence in Human Resource Development: An umbrella review protocol**

**Additional Editor Comments:** I have read the most recent version of the manuscript and I think it addresses the reviewers' comments. Based on reviewers' comments and my own reading of your manuscript, I think your paper have shown a reasonable review protocol. There is, however, a final minor detail regarding your manuscript that will be of great value for our readers. In the last version of the manuscript, you mentioned: "R will be utilized for statistical analysis and visualization." This statement is quite generic and provides no clear guidance. Please be specific regarding the libraries or packages you are going to use. For example, if you plan to do some data visualizations, be aware you can use standard libraries such as R base, or more specialized libraries such as "ggplot2" or "ggstatsplot." Likewise, some analyses can take advantage of using network data such as "igraph" or "statnet" (for authors co-citations analysis) and/or textual data by using packages such as "quanteda" or "tidytext." Be aware that some of these data analysis and data visualizations can be easily achieved with "bibliometrix" and its shiny app "biblioshiny." A short statement illustrating these intended resources can be achieved by including one or two sentences with some of these details. Once these inclusions are addressed, the manuscript can be accepted.

**Responses:** Thank you for the suggestion to specify the packages we plan to use in the analysis stage. As this study involves only descriptive statistics and visualizations, we plan to use R base and ggplot2 for this project. We’ve added a sentence to specify the packages on page 10 of the main protocol:

“R will be utilized for statistical analysis and visualization. We plan to use the base package (R Core Team, 2024) for statistical analysis and ggplot2 (Wickham, 2016) for visualization” (p.10).

It should be noted that, as stated in the data synthesis section, we plan to primarily use thematic coding by employing an HRD framework. While we will definitely consider using SNA, text analysis, and bibliometrics for future potential projects, we do not plan to use them in the current project using this protocol.

**Journal Requirements**: Please review your reference list to ensure that it is complete and correct. If you have cited papers that have been retracted, please include the rationale for doing so in the manuscript text, or remove these references and replace them with relevant current references. Any changes to the reference list should be mentioned in the rebuttal letter that accompanies your revised manuscript. If you need to cite a retracted article, indicate the article’s retracted status in the References list and also include a citation and full reference for the retraction notice.

**Responses:** We have reviewed the reference lists in the main protocol and the supplementary file (S2_File.docx) and confirmed that there are no retracted articles. Therefore, no changes are needed to the reference lists.
